# A Rare Case of Internal Jugular Venous Malformation Treated by Surgical Excision

**DOI:** 10.14797/mdcvj.1336

**Published:** 2024-06-11

**Authors:** Rishik Puppala, Bright Benfor, Shri Timbalia, Tiffany G. Sheu, Alan B. Lumsden

**Affiliations:** 1 Houston Methodist Hospital, Houston, Texas; 2 Texas A&M University, Houston, Texas, US; 3 Houston Methodist Hospital, Houston, Texas, US

**Keywords:** internal jugular venous malformation, IJVM, venous malformation, vascular malformation, IJV, internal jugular vein

## Abstract

This paper reports a case of an internal jugular venous malformation (IJVM) and route of treatment in a patient with limited symptoms. After history and imaging studies, a determination of surgical excision was made to rule out possible malignancy and future problems such as thrombosis. The mass was resected, and part of the IJVM was ligated. The mass had no identifiable malignancy, and the patient recovered fully with no complications. The paper highlights the importance of identifying venous malformations and highlights the reasoning behind the course of action.

## Introduction

Venous malformations are uncommon occurrences that can have a variety of clinical presentations depending on the location.^1^ Malformations are typically congenital^2,3^ and affect about 1% of the population, with an incidence estimated at 1 to 2 per 10,000.^4^ The head and neck are by far the most frequent locations, accounting for as high as 65% of all congenital vascular malformations.^5^ Differentiating between a true venous malformation and a hemangioma entails a thorough history and imaging studies.^4^ Treatment depends on clinical manifestation and ranges from nonoperative management, with observation only, to surgical treatment involving complete excision. In this paper, we report the rare case of a left internal jugular malformation (IJVM) that was treated by surgical excision.

## Case Presentation

### Clinical Presentation

**Figure 1:**
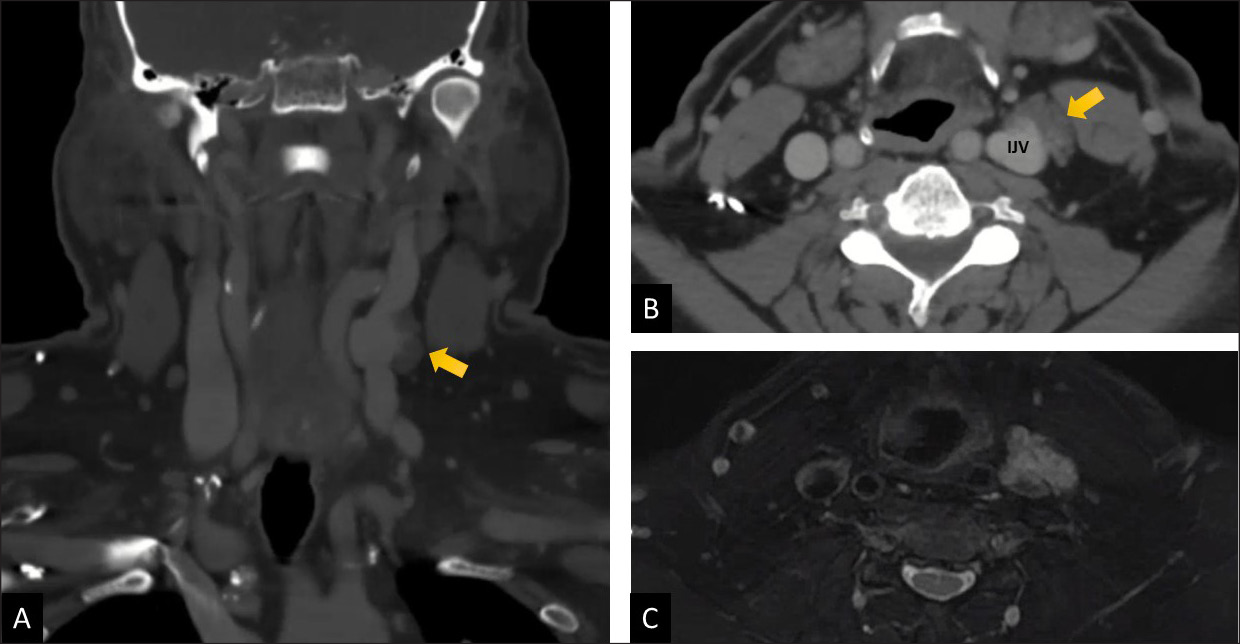
Preoperative imaging of internal jugular venous malformation. **(A)** Coronal view of computerized tomographic scan. **(B)** Axial view of computerized tomographic scan. **(C)** Magnetic resonance imaging showing hyperintensity of lesion in T2.

A 70-year-old male with past medical history of hypertension, diabetes, hyperlipidemia, gastroesophageal reflux disease, and tobacco presented to an outside institution with complaints of neck and shoulder pain. The physical exam at the time was noncontributory apart from tenderness in the right neck and shoulder. A computerized tomographic (CT) scan of the soft neck tissue revealed an incidental left neck mass, which prompted referral to our institution for further management. The CT scan (Figure 1A, B) demonstrated a well-defined left carotid sheath mass that was concerning for a possible schwannoma or lymph node enlargement. This was confirmed on magnetic resonance imaging, which demonstrated a lobulated lesion that was hyperintense in T2 (Figure 1C) and had homogenous enhancement with contrast injection; in addition, it showed that the mass remained inseparable from the ventral surface of the left internal jugular vein. The differential diagnosis at this point was a venolymphatic malformation. However, due to uncertainty regarding the true etiology of the lesion and the possibility of a malignancy, the decision was made to admit the patient for surgical exploration of the neck and eventual biopsy.

### Intraoperative Details

**Figure 2:**
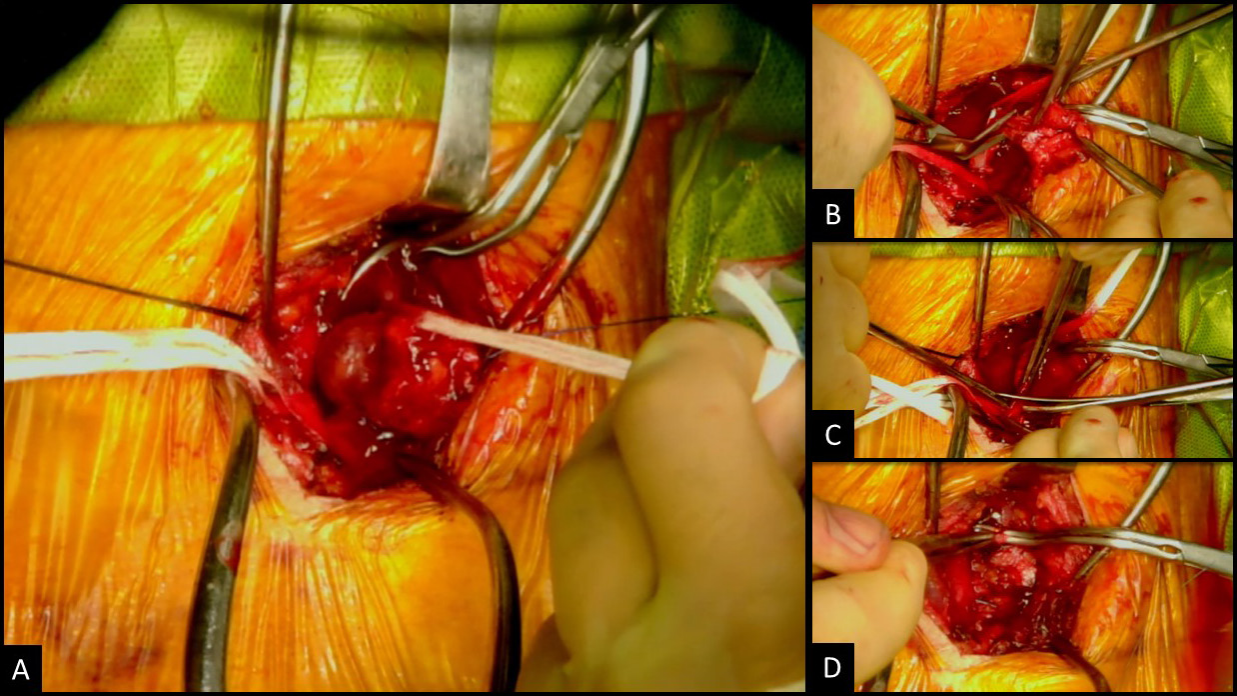
Surgical excision of internal jugular venous malformation. **(A)** Venous malformation adherent to internal jugular vein. **(B)** Excision of malformation. **(C)** Partial resection internal jugular vein. **(D)** Ligation of internal jugular vein stumps.

The procedure was performed under general anesthesia. A 6-cm incision was made along the anterior border of the left sternocleidomastoid and a dissection was carried down to the carotid sheath. The mass was then identified within the sheath on the anterior surface of the IJVM (Figure 2A). It was noted to be highly friable and vascularized. Proximal and distal control of the IJVM was obtained using umbilical tape. The mass was then resected and sent to pathology (Figure 2B). A remnant of the mass was still noted within the wall of the IJVM. Since the patency of the contralateral IJV was confirmed in the patient’s CT scan, the decision was made to resect the portion of the vein that was in contact with the mass (Figure 2C). The proximal and distal stumps were then oversewn with 4-0 Prolene sutures. Hemostasis was achieved using electrocautery and hemoblast, and the wound was closed in layers (Figure 2D).

### Postoperative Course

**Figure 3:**
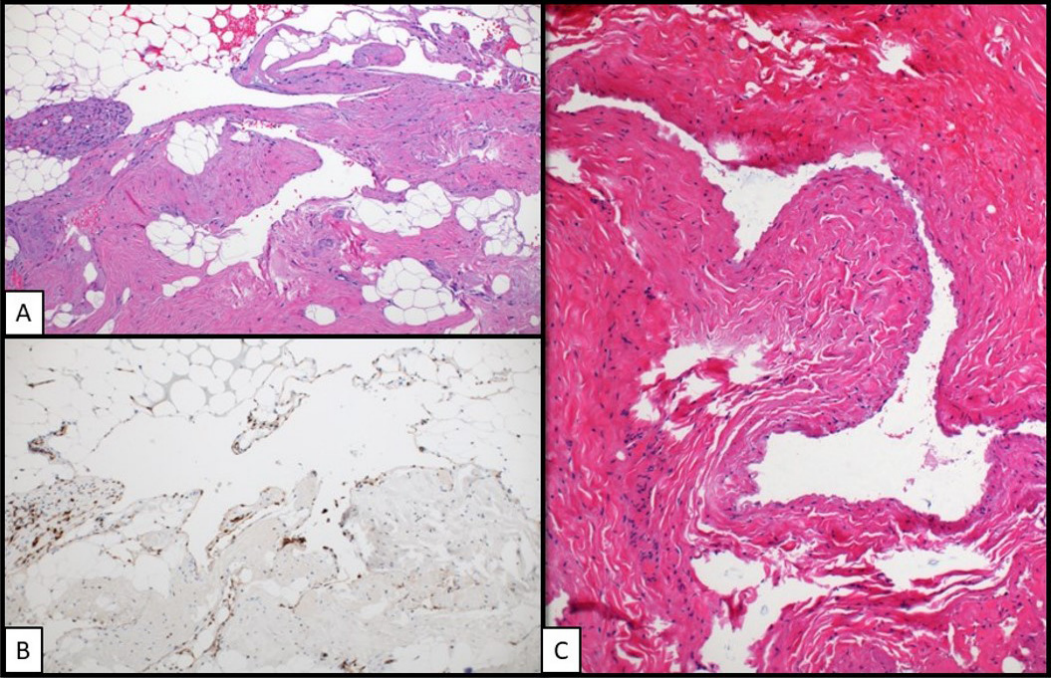
Histopathology analysis of resected lesion (40× magnification). **(A)** H&E stain of vascular malformation. **(B)** Immunohistochemical stain highlighting endothelial cells of vascular malformation. **(C)** H&E stain of left internal jugular vein.

The postoperative course was uneventful, and the patient was discharged home the next day on pain medication. Pathologic evaluation demonstrated a vascular malformation associated with a medium-sized venous malformation (Figure 3A-C). No malignancy was identified. The patient was doing well upon 2-week and 6-month follow-up, with no reported issues on the operated side and no further complaints of the initial right-sided neck pain.

## Discussion

This paper details an incidental IJVM discovered during imaging for right shoulder and neck pain. Internal jugular venous malformation remains extremely rare and usually presents as asymptomatic soft swelling in the neck, with slow growth.^1^ On the other hand, it may be associated with pain depending on the size, extent, and mass effect.^6^ The diagnosis relies on Doppler flow imaging and magnetic resonance imaging, which help to differentiate a venous malformation from other vascular malformations and possible malignancy.^4,7^

Due to their rarity, there are currently no standardized guidelines for the management of IJVMs.^8^ Current indications for treatment are geared towards IJVMs causing any functional limitation or pain in the patient.^9^ Nonsurgical therapeutic treatments include compression garments and sclerosing agents.^9^ Additionally, if there is no limitation or pain upon discovery of a vascular malformation, no intervention may be needed.^10^ In this present case, surgical intervention was mostly indicated to establish the etiology of the lesion due to concerns for a possible malignancy.

Vascular malformations in the head and neck may be complicated with venous thrombosis or vascular compression that restricts blood flow to and from critical structures and, therefore, would require more thorough examination.^8^ One major concern in our present case was the retention of malformation tissue within the wall of the IJV, which we feared could lead to thrombosis down the line. This, coupled with the uncertainty surrounding its etiology, informed our decision to partly resect the IJV. We opted not to reconstruct the IJV since studies have shown that unilateral IJV ligation is relatively safe and not associated with significant clinical impairment.^11,12^ However, it is essential to ensure the contralateral IJV is intact prior to any ligation.^11,12^ The lack of standardized guidelines for the treatment of vascular malformations creates a need for discretion on the part of the surgeon. We have learned through our extensive experience that, in most cases, a more aggressive surgical approach was effective in treating the malformation, and noninvasive treatments in this area would not ameliorate symptoms nor address the concern for future complication.^13^ It is always important to consider the symptoms and location to determine if surgical excision is the best treatment. Further studies are required to elucidate the optimal management algorithm for IJVMs.

## Conclusion

Internal jugular vein malformations are rare and currently have no standardized treatment. Based on a thorough history and imaging studies, a determination of how to proceed with treatment can be made. In symptomatic or highly problematic cases, surgical excision is the best course of treatment to prevent further complications downstream.
